# Nanoscale mapping of composition and orientation in electrospun polymeric nanofibers loaded with carbon atomic wires

**DOI:** 10.1038/s41598-026-46988-8

**Published:** 2026-04-04

**Authors:** Simone Melesi, Devon S. Jakob, Jeremy F. Schultz, Adam J. Biacchi, Piotr Pińkowski, Bartłomiej Pigulski, Chiara Castiglioni, Chiara Bertarelli, Sławomir Szafert, Carlo S. Casari, Andrea Centrone

**Affiliations:** 1 Micro and Nanostructured Materials Laboratory — NanoLab, Department of Energy, Politecnico di Milano, Via Lambruschini 8, Milano, 20156 Italy; 2 Nanoscale Device Characterization Division, Physical Measurement Laboratory, National Institute of Standards and Technology, Gaithersburg, Maryland, 20899 USA; 3 Faculty of Chemistry, University of Wrocław, 14F. Joliot-Curie, Wroclaw, 50–383 Poland; 4 Department of Chemistry, Materials and Chemical Engineering “Giulio Natta”, Politecnico di Milano, Milano, Italy

**Keywords:** Carbyne, Electrospinning, Orientation, AFM-IR, O-PTIR, Hyperspectral Photoluminescence, Materials science, Nanoscience and technology, Optics and photonics

## Abstract

This study yields a comprehensive nanoscale investigation of the composition and molecular orientation of electrospun poly(methyl methacrylate) (PMMA) nanofibers containing halogen-terminated carbon atomic wires (CAWs), a finite-length analog of the carbyne construct. This is accomplished by conducting light-polarization-dependent experiments with advanced techniques such as atomic force microscopy-infrared spectroscopy (AFM-IR), optical photothermal infrared microscopy (O-PTIR), and hyperspectral photoluminescence (PL) microscopy. In PMMA nanofibers, AFM-IR reveals a remarkable, homogeneous uniaxial orientation of PMMA chains along the fiber axis, down to the ≈ 20 nm scale. Semi-quantitative analysis of IR dichroic ratios indicates a nearly orthogonal alignment ($\:{\psi\:}_{avg}\approx\:\:79^\circ\:$) of the C = O dipole with respect to the nearly all-trans planar polymer backbone, consistent with expectations of the electrospinning process and in agreement with prior modeling of polymer chain dynamics. Congruous AFM-IR, O-PTIR, and hyperspectral PL data show that incorporation of CAWs into the PMMA nanofibers results in a peculiar compositional heterogeneity, with alternating 3 μm to 4 μm long PMMA-rich and CAW-rich regions across the fiber length. Notably, hyperspectral PL data reveal that CAWs preferentially align along the fiber axis, with sparse, 1.5 μm to 3 μm long domains displaying locally reduced or enhanced molecular orientation. We envision that the widely applicable approach used here will foster engineering of anisotropic nanostructures with advanced functionalities and complex compositions, laying the groundwork for future applications in nanoelectronics, photonics, and energy storage that demand anisotropic molecular orientation and composition.

**Supplementary Information:**

The online version contains supplementary material available at 10.1038/s41598-026-46988-8.

## Introduction

Carbynes are infinitely long, one-dimensional chains of sp-hybridized carbon atoms^1,2^. They attract growing interest thanks to mechanical, electrical, and optical properties that have been predicted to outperform those of graphene, carbon nanotubes, and other carbon nanostructures^3–10^. Carbon atomic wires (CAWs), the finite-length experimental realization of the carbyne construct (Fig. S1), have shown promise as additives in advanced nanocomposite materials^11^ and as active elements in field effect transistors, organic electronics, and supercapacitors^12–14^.

The one-atom-diameter structure of CAWs is strongly anisotropic. For example, first-principles calculations predict record-breaking stiffness along the carbynes’ chain axis^3,11^. Similarly, optical properties such as absorption^15,16^, photoluminescence^15,17^, dielectric function^18^, first-order hyperpolarizability^19^, and transport properties, e.g., current and electron density^3,20^, are also anisotropic and considerably enhanced along the chain axis. However, the engineering of devices and composites harnessing such directionally outstanding properties requires new methods for assessing and controlling the alignment of CAWs. Thus far, only a few studies have reported aligned CAWs. For example, CAWs stabilized by gold clusters were aligned with an applied electric field^21,22^ or by gravity^15^, while hydrogen-terminated CAWs were aligned within a polyvinyl alcohol (PVA) matrix by uniaxial stretching^16,17^. Recently, electrospinning has been proposed as an alternative strategy to incorporate CAWs within poly(methyl methacrylate) - PMMA nanofibers^23^. Since electrospinning produces nanofibers^24–27^ by ejecting a polymeric solution from a needle under high bias, the polymeric chains experience intense stretching forces that induce molecular alignment^28–31^. Commonly, a suitable substrate collector (e.g., rotating drum, wheel-like bobbin, split electrode, etc^27–33^, is employed to obtain nanofibers with a high degree of macroscopic geometrical alignment. While the alignment of polymeric macromolecules has been extensively investigated at a macroscopic scale (i.e. averaging the orientation of many fibers) these effects are largely unexplored at the single or intra-fiber levels and not yet reported for CAWs in polymeric fibers. Although a certain degree of orientation of polymer chains within an electrospun nanofiber should be expected, the orientation of short, additive, molecules like the 4-carbon-long molecules studied here, in such fiber composites is neither expected nor has been reported previously. Given CAWs’ finite length, understanding their physical aggregation within a host medium, like the fibers in our samples, is critically important to establish percolative paths of electronic transport^38^ or to exploit anisotropic effects in hetero-terminated dipolar or push-pull carbon wires^23^.

Here, a suite of high spatial resolution spectroscopic techniques, such as atomic force microscopy infrared spectroscopy (AFM-IR)^32–35^ and hyperspectral photoluminescence (PL) microscopy^36,37^ are used to determine the composition and orientation heterogeneity of PMMA nanofibers containing halogen-terminated CAWs^38,39^. The AFM-IR technique, also known as photothermal induced resonance (PTIR), combines the chemical specificity of IR spectroscopy with the spatial resolution of atomic force microscopy (AFM), enabling IR analysis at the ≈ 10 nm scale^38^; i.e., much smaller than the optical diffraction limit. Spectra obtained with AFM-IR/PTIR are typically characterized by undistorted, absorptive profiles^39,40^ which are congruous with the spectra in far-field IR databases, leading to a steadily growing number of applications encompassing material science^41–44^, photovoltaics^45,46^, medicine^47–52^, geology^53,54^, and art conservation^55,56^ among others. Recent PTIR experiments with wide-bandwidth optomechanical AFM probes^57–59^ also enabled quantitative measurement of the thermal properties of materials at the nanoscale. Moreover, control over the polarization of light used in AFM-IR measurements has proven useful in the study of anisotropic materials, enabling direct study of their molecular orientation^60,61^. We note that AFM-IR is one of several nanoscale IR techniques, including scattering-type scanning near-filed microscopy (s-SNOM)^35^, peak-force tapping IR (PFIR)^62^ and photoinduced force microscopy (PiFM)^63^. Details on operating principles and applications are available in recent reviews^32–35,64^.

The rapid analysis of hyperspectral PL microscopy based on high-throughput imaging filters has also enabled broad applications in material science^36^, and photovoltaics^37^ and has been shown to complement AFM-IR for photoluminescent samples^36^.

In this study, AFM-IR experiments with polarized light reveal remarkable uniaxial intra-fiber orientation of polymeric chains in PMMA nanofibers along the fiber direction that is homogeneous at the ≈ 20 nm scale. In contrast, AFM-IR data obtained on CAW-loaded PMMA nanofibers show chemical heterogeneity with CAWs enrichment in regions with locally larger fiber diameters. These results are corroborated at the ≈ 500 nm scale using the optical photothermal infrared technique (O-PTIR)^65^. However, the AFM-IR and O-PTIR signal intensities are insufficient to estimate the local orientation of CAW-rich regions. Remarkably, complementary polarized hyperspectral PL data clearly show that CAW molecules are primarily oriented with their molecular chain axis parallel to the fiber axis at the ≈ 500 nm scale, demonstrating that electrospinning-induced forces promote alignment of CAW molecules within nanofibers. Therefore, although the short (four sp-hybridized carbon atoms long) CAWs employed here differ from the ideal (i.e. infinite length) carbyne model, they can be engineered to exhibit anisotropic properties, highlighting the effectiveness of electrospinning for producing anisotropic nanocomposites.

In summary, the advanced characterization described in this work provides a detailed, spatially resolved picture of the internal structure and organization of nanocomposite systems. Such structural information encompasses the orientation of polymer chains, phase separation and spatial distribution of constituents, and the alignment of domains within individual nanostructures. Although this proof-of-principle-investigation focuses on the structural and molecular characterization of our samples, we believe that the wide applicability of our approach will foster future engineering efforts for realizing anisotropic nanostructures with advanced functionalities and complex compositions.

## Experimental section

Poly(methyl methacrylate) (PMMA) with M_w_ = 996k and high-performance liquid chromatography grade (≥ 99.7%) N, N-dimethylformamide (DMF) were used as received. Halogenated CAWs (referred to as C_4_I) were synthesized following a previously reported procedure^66–68^. The chemical structure of C_4_I is shown in Fig.S1.

PMMA and PMMA/C_4_I nanofibers were produced by electrospinning a PMMA 6% g/g mass fraction (0.6 M, based on the monomer molecular mass) solution in DMF and a 6% mass fraction solution of PMMA in DMF with the addition of C_4_I (0.36 M), respectively^23^. Briefly, PMMA was stirred in DMF at 60 °C until completely dissolved (≈ 5 h). After cooling down to room temperature, the C_4_I powder was added to the polymeric solution and stirred for 3 h until a homogeneous solution was obtained. Electrospinning was performed in a horizontal configuration by loading the feed solution in a 2.5 mL syringe with a 22-gauge needle and using an infusion pump. Aligned nanofibers were obtained by electrospinning the solutions for 5 s onto Si substrates (P-doped with low IR absorption) mounted on a rotating drum (1200 min^− 1^) with a needle-to-collector distance of 20 cm by applying 12 kV (DC) to the needle and using 0.2 mL/h rate. A schematic representation of the electrospinning setup is reported in the Fig. S2. After DMF evaporation via electrospinning deposition and considering the initial concentrations of the constituents in the solution, the nominal mass fraction of C_4_I within the nanofibers is expected to be 62.5%.

The diameter distribution of the fibers and their relative orientation were determined from images obtained using a field emission scanning electron microscope (FE-SEM) and an image processing software by analyzing approximately 100 fibers. FE-SEM images on as-prepared (uncoated) samples were acquired in low-current mode, using an acceleration voltage of 5 kV, a vacuum pressure in the sample chamber of ≈ 0.5 Pa, and magnifications ranging from 10000x to 100000x.

AFM-IR experiments were obtained using modified, commercially available setup which has been described recently^38^. Briefly, mid-IR light pulses from a quantum cascade laser (QCL) were focused at a ≈ 20º angle (with respect to the sample surface) to a ≈ 50 μm diameter spot on the sample surface centered around a gold-coated silicon AFM probe tip (0.07 N/m to 0.4 N/m nominal spring constant, 13 kHz ± 4 kHz nominal free-space oscillation frequency, ≈ 25 nm nominal tip radius). A pair of precisely calibrated beam-steering mirrors maintained the laser spot position relative to the AFM probe at all wavelengths. All AFM-IR data were obtained with a QCL emitting linearly polarized light between 910 cm^− 1^ to 1905 cm^− 1^, unless otherwise noted. A polarization rotation device was used to select either s- or p- light polarization illumination at the sample^38^. Given the weak IR signal originating from the carbyne component, the carbyne loaded fibers were measured with a resonant excitation scheme^33,69^ for increased sensitivity. The laser repetition rate was matched to one of the AFM probe mechanical resonances (e.g. ≈ 460 kHz). We note that while the resonant excitation gain yields stronger signals, the gain depends on the local mechanical properties of the sample, i.e. its stiffness (causing resonance shifts) and damping (changing the resonance *Q*)^33^. A phase-locked loop^70^ was employed to maintain the resonance excitation condition which, however, compensates only for resonance shifts and not variations of Q. Therefore, quantitation of AFM-IR data recorded on resonance are typically more challenging.

For unloaded PMMA fibers, we employed an off-resonant excitation^40^ by fixing the laser repetition rate to 250 kHz, which is not a mechanical mode of the AFM probe, and demodulating at the same frequency. Such off-resonance detection scheme was chosen to facilitate quantitative analysis of AFM-IR data as it is largely independent from, locally varying, tip-sample interactions and sample mechanical properties during a scan^40^. Spectra (1 cm^− 1^ resolution) were obtained by holding the probe stationary while sweeping the laser wavelength at 50 cm^− 1^/s and referencing the polarization specific laser output intensity (background spectrum) measured with a pyroelectric detector. This was necessary because of the polarization dependent reflectivity of the protected silver mirrors used in our setup. AFM-IR absorption maps were obtained by fixing the laser excitation wavelength and scanning the AFM probe (0.15 Hz). While AFM-IR spatial resolution can be < 10 nm^38^, the spatial resolution in this work was limited by the pixel size, as described in each caption.

Hyperspectral PL images (550 nm to 740 nm, 4 nm spectral resolution) were collected at a rate of 0.1 nm/s using a microscope equipped with a high-throughput Bragg grating filter and coupled to a complementary metal-oxide semiconductor (CMOS) camera (2048 × 2048 pixels array, 82% peak quantum efficiency, 37 000:1 dynamic range). A linearly polarized 1 W 532 nm continuous wave laser was used for widefield excitation through a 100x objective (0.9 numerical aperture, 1 mm working distance). PL emission was collected through the same objective with a linear polarizer positioned parallel to the excitation orientation. Polarized hyperspectral PL was conducted with excitation and analysis orientation either parallel or perpendicular to the fiber axis. The resulting images had a pixel resolution of ≈ 65 nm with a spatial resolution of < 1 μm. PL spectra were normalized by the dark background intensity. Spectral profiles were generated by plotting emission intensity at 602 nm as a function of position along the center of the fiber.

## Results and discussion

**Fig. 1:**
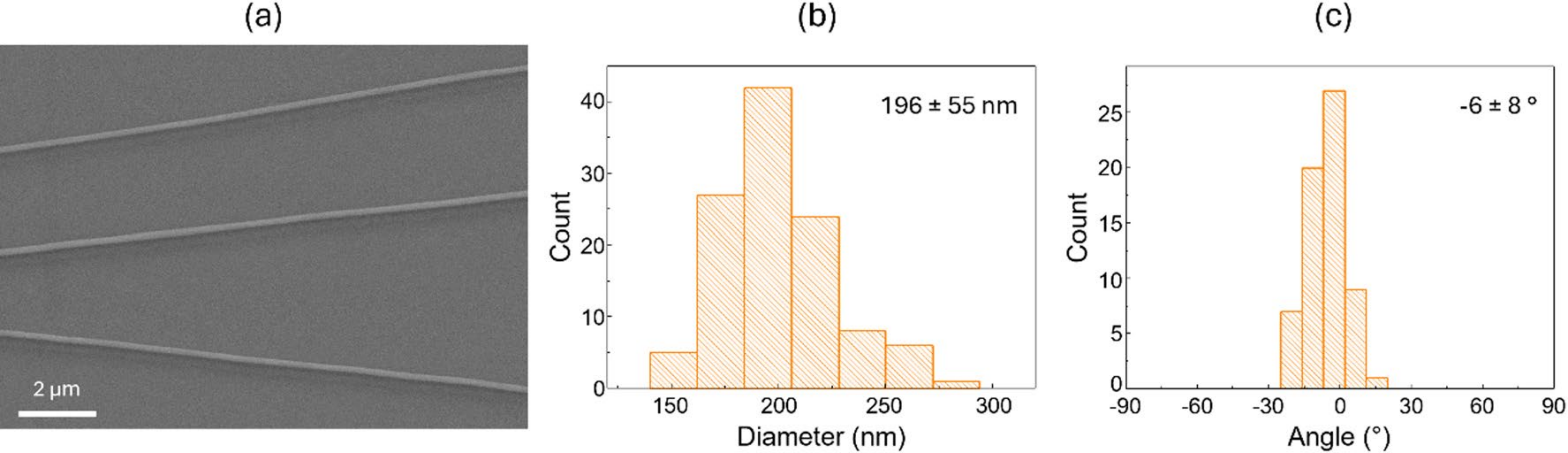
(**a**) SEM image of CAW-loaded PMMA electrospun nanofibers. (**b**) Diameter distribution of the nanofibers. (**c**) Geometrical orientation distribution highlighting their degree of alignment.

Analysis of representative SEM images of CAW-loaded PMMA electrospun nanofibers (Fig. 1a) show a proper morphology, meaning that the fibers are continuous, uniform, and smooth, with no evidence of common electrospinning defects such as beads, surface irregularities, or fused/agglomerated regions. These features indicate that the processing parameters yielded well-defined nanofibers with a controlled structure. The fibers average diameter was 196 nm ± 55 nm; see Fig. 1b. Throughout the paper, the reported uncertainties are one standard deviation statistical uncertainty, unless noted otherwise. The rotating drum collector (see Experimental Methods) enables the production of electrospun nanofibers with high degree of macroscopic orientation. Specifically, the degree of alignment—expressed as the angular deviation, i.e., the standard deviation from the average macroscopic orientation— was just 8°. The distribution of the angles is reported in Fig. 1c.

The AFM-IR technique was employed to gain insights into the fibers’ composition and molecular orientation details at the nanoscale. In AFM-IR, the probe tip of an AFM measures absorption spectra and maps with ≈ 10 nm spatial resolution^38^ by transducing the photothermal expansion of a sample due to the absorption of short laser pulses. The fast photothermal expansion of the sample excites the AFM probe motion, inducing oscillations at its mechanical resonances with amplitudes proportional to the sample’s local optical absorption coefficient^38,39,58,71,72^. Notably, in contact mode, a defining characteristic of AFM-IR is the ability to probe the composition of a sample at depth exceeding 1 μm^72^. Since AFM-IR depth sensitivity exceeds the thickness of the samples analyzed in this work (≈ 200 nm to ≈ 500 nm) it provides local compositional and orientational properties averaged through the fiber thickness. This differs from other commonly used AFM-based IR techniques, such as s-SNOM^35^ and PiFM with sideband excitation^73^ which strongly leverage the tip near-field and have a near-surface depth sensitivity. We note that PiFM with direct mode excitation was also reported having depth sensitivity up to ≈ 1 μm^74,75^.

**Fig. 2:**
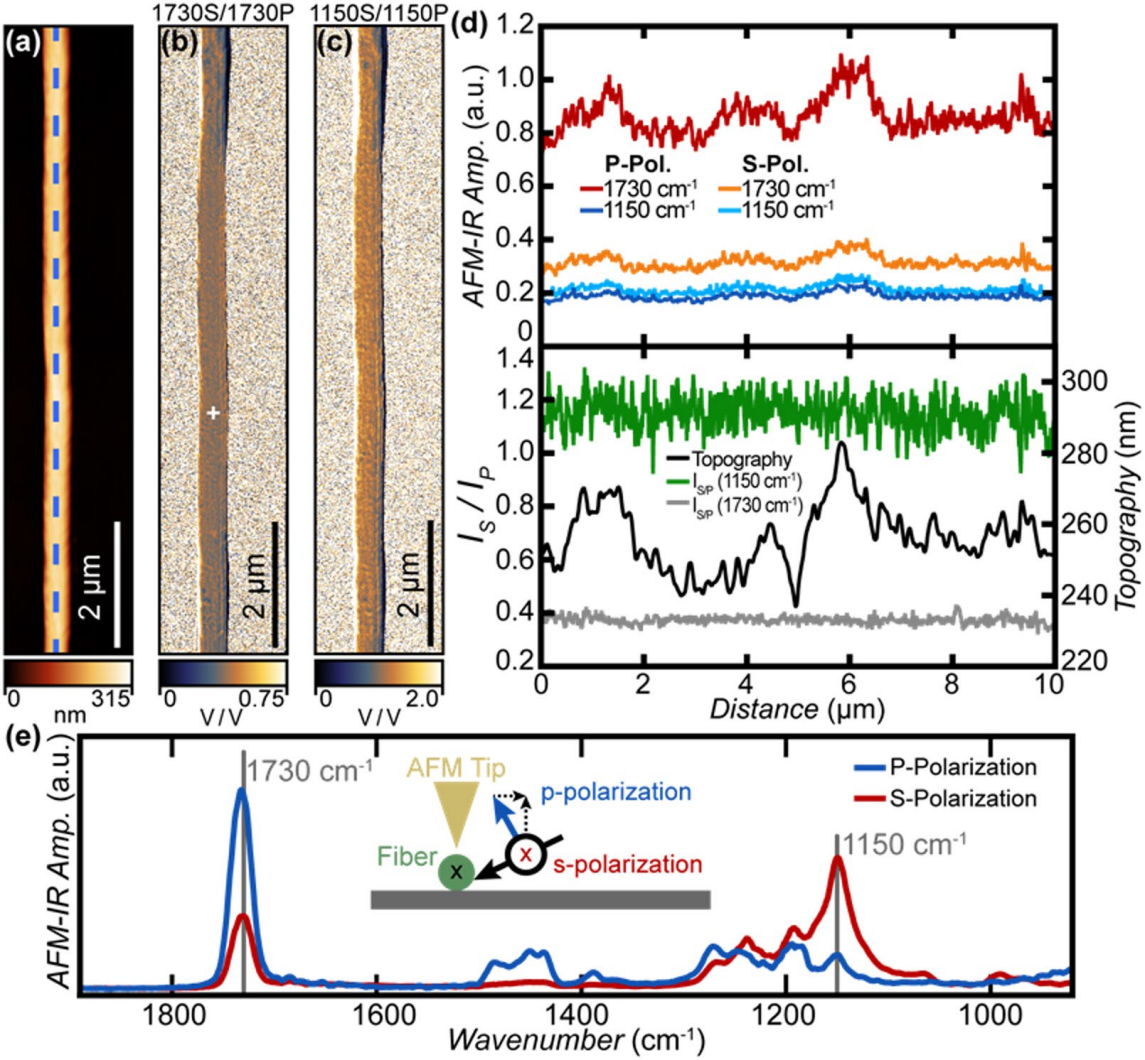
(**a**) AFM topography image and corresponding AFM-IR absorption ratio images (9.8 nm x 15.6 nm pixel resolution in the vertical and horizontal direction, respectively) measured with s- and p-polarization (normalized for the wavelength-dependent laser power) at characteristic PMMA absorption peaks: (**b**) 1730 cm^− 1^ and (**c**) 1150 cm^− 1^. The homogeneity of the AFM-IR image ratio maps suggests that the PMMA fiber is characterized by a homogeneous intra-fiber molecular orientation. (**d**) Top: common-scale AFM-IR absorption intensity profiles obtained at 1730 cm^− 1^ (red, orange), and 1150 cm^− 1^ (blue, light blue) for p- and s-polarization, respectively along the fiber axis in correspondence to the line marked in panel (a). Bottom: AFM topography (black), AFM-IR intensity ratio (s-/p-polarization) at 1730 cm^− 1^ (gray) and 1150 cm^− 1^ (green) along the fiber axis in correspondence to the line marked in panel (a). (e): representative normalized and common scale AFM-IR absorption spectra obtained at the marked location in panel (b) for s- (red) and p-polarization (blue). The strong variation of relative intensity for s- and p-polarization spectra is suggestive of strong intra-molecular alignment within the fiber. The inset schematic shows that for s-polarization (red) the polarization of light is parallel to the fiber axis while for p-polarization (blue) the light polarization has two components that are both perpendicular to the fiber axis. For p-polarization, in AFM-IR the gold coated AFM tip typically provides an enhancement (≈ 2.2x in this case) for the vertical field intensity component within the tip-near field^33,69^.

First, we assess the composition and orientation of unloaded (i.e., PMMA only) nanofibers (Fig. 2). A custom rotation stage was used to align the direction of the fibers to be approximately parallel with the long axis of the AFM cantilever to better study their molecular orientation. Given the 20° incident angle, the s-polarization configuration results in light polarization aligned with the long axis of the fiber (Fig. 2e inset, red). In contrast, when p-polarization is selected (see Fig. 2e inset, blue), two polarization components (in and out of the sample plane) excite the sample, both perpendicular to the fiber axis. Given the cylindrical symmetry of the fiber geometry and the reasonable hypothesis of uniaxial fiber orientation imparted by the electrospinning process, the two orthogonal p-polarized field components add up to a pure perpendicular component. We note, however, that in the case of p-polarization, the vertical (out-of-plane) component could be enhanced in the tip near-field^69^ for the top portion of the fiber. Figure 2a shows a representative AFM topography image, while the corresponding AFM-IR absorption images at 1730 cm^− 1^ and 1150 cm^− 1^ (PMMA carboxylic stretching and skeletal stretching modes, respectively) are reported in Fig. S3, for both s- and p-polarizations. The topography profile (Fig. 2d) extracted along the center of the fiber highlights local variations in the fiber thickness (i.e., diameter) up to ≈ 40 nm. The corresponding AFM-IR intensity profiles in Fig. 2d show stronger absorption in regions with locally larger thicknesses at all measured frequencies. This is typical for AFM-IR since the amount of light locally absorbed by the sample and the measured signal are proportional to the sample absorption coefficient and to the amount of material under the tip^33,39,40,72,76^.

To determine the molecular orientation of the fibers, we consider the intensity ratios of AFM-IR spectra (Fig. 2e) and maps (Fig. 2b, c) and the relative orientation of IR-active molecular vibrations localized on specific chemical groups with respect to the fiber axis. Representative AFM-IR absorption spectra with s- (red) and p-polarization (blue) are shown in Fig. 2e. To enable quantitative analysis, the spectral intensities in Fig. 2e were normalized with respect to the polarization dependent intensity (i.e., background, see Fig. S4) and with respect to the estimated tip enhancement. Since the depth of the enhanced near-field is comparable to the AFM tip radius (≈ 25 nm) and since, in contact-mode, AFM-IR probes samples at depths exceeding 1 μm^40^, for our sample (≈ 200 nm thick) the relative effect of the near-field tip amplification is generally weak. Since it is not possible to use the sample vibrational peak intensity to estimate an unknown tip enhancement for a sample of unknown molecular orientation, the tip enhancement with p-polarization (E_tip_ ≈ 2.16 ± 0.14) was estimated here as the ratio of the average p- and s-polarization intensities in a nominally non-absorbing region of the spectra (i.e., 1810–1880 cm^− 1^). The uncertainty of the tip enhancement estimation corresponds to a single standard deviation and is primarily determined by the noise baseline in the recorded data.

The strong variation of relative peak intensities in the s- and p-polarization spectra indicates strong intra-molecular alignment of PMMA molecules within the fiber. This is not surprising since the polymer chains stretch in the jet during the electrospinning process and often result in a preferential uniaxial orientation with polymer chain axes mostly aligned parallel to the fiber axis^28–31^. However, the spectra in Fig. 2e and the image ratios in Fig. 2b, c encode information regarding the fiber orientation that is local (i.e. with ≈ 20 nm lateral resolution) and averaged with respect to the local thickness of the fiber^40^.

Since AFM-IR signal transduction and its signal intensities are also scaled by the sample local mechanical properties, rather than using single wavelength absorption images, it is common to assess the variation of composition or orientation using image ratios, which in first approximation are independent from variations of mechanical properties in the sample^33,48,77^. Similar to the spectra, the intensities of image ratios in Fig. 2b, c were scaled based on the background power intensity and estimated tip enhancement. The homogeneity of s/p AFM-IR image ratios in Fig. 2b (1730 cm^− 1^, carboxylic stretching mode) and Fig. 2c (1150 cm^− 1^, skeletal stretching mode) see also the image ratio longitudinal cuts in Fig. 2d, indicates that the local molecular orientation within the PMMA fiber is generally homogeneous, The very narrow dark blue and bright white regions at the right and left sides of fiber in the image ratios in Fig. 2b, c) are interpreted as an artifact due to sample drift occurring while acquiring consecutive images. We note that the s-/p-polarization intensity ratio at 1730 cm^− 1^ from the spectra in Fig. 2e (≈ 0.40) matches well the values from the corresponding line cut in Fig. 2d (0.37 ± 0.01) but that at 1150 cm^− 1^ the value from the linecut in Fig. 2.d (≈ 1.2) is lower than for the spectra in Fig. 2e. (≈ 4.0). While we are not sure of the origin for such discrepancy, data quantitation throughout the manuscript is carried out using the 1730 cm^-1^ peak.

Having established that the orientation of PMMA molecules in the fiber is homogeneous within the fiber, next we infer their average orientation using the dichroic ratio (R = A_s_/A_p_) for the PMMA C = O stretching ($\:{R}_{1730}=0.353$) in Fig. 2e:

Under the reasonable hypothesis of uniaxial orientation, the experimental IR dichroic ratio can be used to assess the degree of molecular orientation according to the Herman’s function $\:f\:$^78,79^:

1 $$\:f=\frac{\left(R-1\right)\:({R}_{0}+2)}{\left(R+2\right)\:({R}_{0}-1)}$$

2 $$\:f=\frac{3<{\mathrm{c}\mathrm{o}\mathrm{s}}^{2}\varPhi\:>-1}{2}$$

3 $$\:{R}_{0}=\:2\:{\mathrm{c}\mathrm{o}\mathrm{t}}^{2}\psi\:$$

**Fig. 3:**
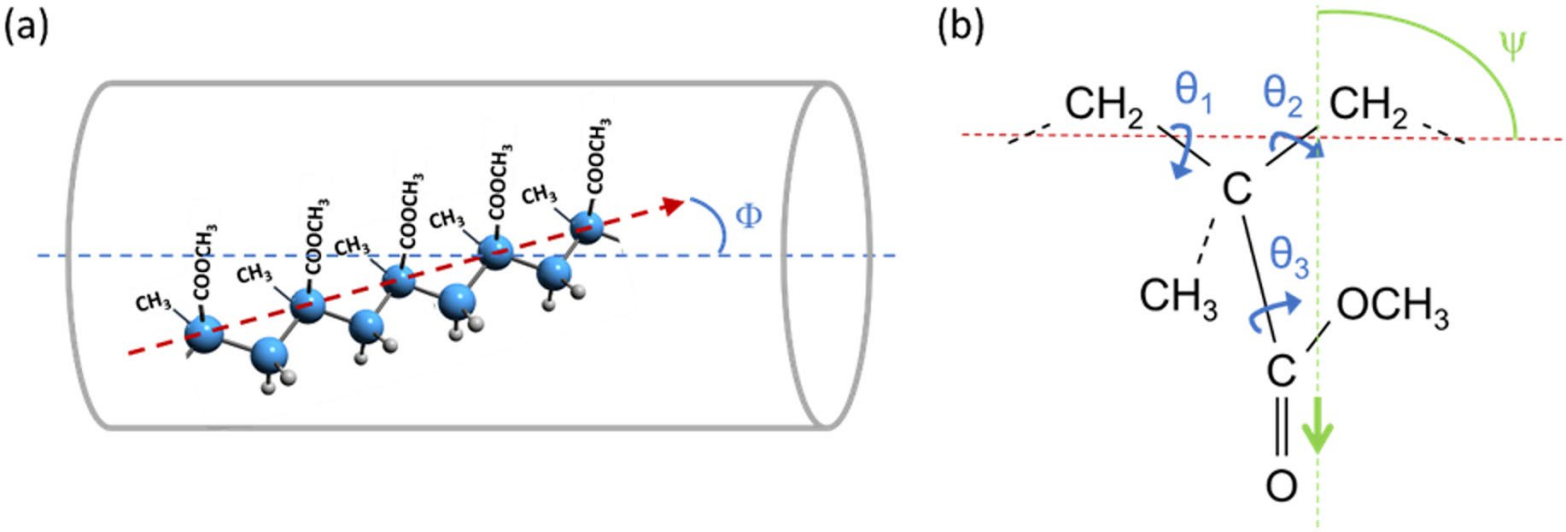
(**a**) Schematic (not to scale) highlighting the angle Φ between the polymer chain axis (red line) and the fiber axis (director, blue line). The fiber is represented by the cylinder. (**b**) Sketch of PMMA repeating unit for the polymer backbone in all trans conformation and the functional CH_3_-C-COOCH_3_ group in a plane orthogonal to the chain axis (torsional angle θ_3_ = 0°), the most favored configuration according to Behbahani et al.^80^ The dipole moment associated with the C = O stretching mode is represented by the green arrow, which forms an angle ψ with the polymer chain axis (in red). See the Supplementary Information for the full discussion of the PMMA molecular structure.

Where *Φ* is the angle between the polymer chain axis and the fiber axis (see Fig. 3a). While for an amorphous polymer like PMMA, a distribution of *Φ* values shall be expected for the molecules making up the fiber, it is customary to consider an equivalent ideal sample where all the chains have exactly the same angle $\:\stackrel{-}{\varphi\:}=\mathrm{a}\mathrm{r}\mathrm{c}\mathrm{o}\mathrm{s}{\left(<{\mathrm{c}\mathrm{o}\mathrm{s}}^{2}\varPhi\:>\right)}^{1/2}$, so that $\:f=(3{cos}^{2}\stackrel{-}{\varPhi\:}-1)/2$.

$\:{R}_{0}$ is the ratio between the parallel and perpendicular components of the vibrational dipole moment (e.g., of the carboxylic stretch at 1730 cm^− 1^), and the fiber axis, while $\:0\le\:\psi\:\:\le\:\:{\uppi\:}$ is the angle between the direction of the molecular dipole moment vector and the chain axis (see Fig. 3b).

While the assignment of a band to a given vibrational normal mode allows the prediction of the direction of the transition dipole moment and $\:{R}_{0}$ for highly symmetric molecules, for low symmetry polymers like PMMA, $\:{R}_{0}$ is typically unknown. Even so, here we use the experimental $\:{R}_{1730}$ value to determine the orientation of the PMMA chains in the fiber semi-quantitatively, without any assumption on the dipole moment direction (i.e. on the value of *ψ*). See the Supplementary Information for details.

Hence, since $\:0\:\le\:{\mathrm{c}\mathrm{o}\mathrm{s}}^{2}\varPhi\:\le\:1$, according to Eq. 3: $\:-\frac{1}{2}\le\:f\le\:1$ where the limiting cases represent perfect polymer chain orientation either orthogonal ($\:f=-\frac{1}{2}$) or parallel ($\:f=1$) to the fiber axis, respectively.

Given the experimental $\:{R}_{1730}$ = 0.353, the range of mathematically acceptable values for $\:f$ provides intervals for the allowed values for $\:{R}_{0}$, $\:\psi\:$ and $\:\stackrel{-}{\varphi\:}$:

$$\:\mathrm{C}\mathrm{a}\mathrm{s}\mathrm{e}\:1:\:{R}_{0}>1\Rightarrow\:{R}_{0}\ge\:4.666\:;0\le\:\psi\:\le\:33.2^\circ\:;\:-\mathrm{0,5}\le\:f\le\:-0.28;\:90^\circ\:\ge\:\stackrel{-}{\varphi\:}\ge\:67.2^\circ\:$$

$$Case\, 2:\:{R}_{0}<1\Rightarrow\:{0\le\:R}_{0}\le\:0.353\:;67.2^\circ\:\le\:\psi\:\le\:90^\circ\:;\:0.59\le\:f\le\:1;\:31.5^\circ\:\:\ge\:\stackrel{-}{\varphi\:}\ge\:0^\circ\:$$

Case 1 ($\:f$ values close to the limiting case of chains perfectly oriented orthogonal to the fiber axis) can be discarded, as it contrasts with the physics of the electrospinning process that stretches and preferentially orients the polymer chains close to the fiber axis direction.

The acceptable values for $\:\psi\:$ in case 2 range from $\:\psi\:=90^\circ\:$ (corresponding to a C = O stretching transition dipole perfectly orthogonal to the chain axis) to $\:\psi\:=67.2^\circ\:$ (i.e., nearly orthogonal to the chain axis). Interestingly, this corresponds to a small range of acceptable $\:f$ values, close to $\:f=1$, i.e., perfect alignment of the chains in the fiber axis direction and $\:\stackrel{-}{\varphi\:}=0^\circ\:$. The lower limiting value $\:f=0.59$ corresponds to $\:\stackrel{-}{\varphi\:}=31.5^\circ\:$; i.e., the worst possible degree of orientation in the PMMA fibres compatible with the experimental dichroic ratio. This finding agrees with previous molecular dynamics simulations and statistical analysis^80^, which identified the all trans, fully planar, backbone structure and the chemical group CH_3_-C-COOCH_3_ in a plane orthogonal to the chain axis (i.e., $\:\psi\:=90^\circ\:$) as more statistically favored. Considering deviations from an ideal polymer structure (e.g., irregular tacticity, conformational disorder) that should be expected for amorphous polymers and that would yield a distribution of orientations for the C=O bonds with respect to the polymer chain axis, the interval 67.2° ≤ *ψ* ≤ 90 from our experiment appear to be fully acceptable. Given the intrinsic conformational disorder of the PMMA chains we take $\:{\psi\:}_{avg}=78.6^\circ\:$, corresponding to the average $\:\psi\:$ value in the allowed interval, as a reasonable value to describe the direction of the carboxylic stretching with respect to the polymer backbone. This choice gives $\:{f}_{avg}=0.623$ and $\:{\stackrel{-}{\varphi\:}}_{avg}=30.1^\circ\:$, corresponding to a good chain orientation along the fiber. Such analysis is possible because the C=O stretching mode is largely localized on the C = O unit and its transition dipole moment can reasonably be assumed to lie parallel to the C = O bond (Fig. 3b). It would be tempting to conduct analogous analysis for the 1150 cm⁻¹ mode that, based on its polarization response (Fig. 2e), shall have a transition dipole moment with a large component along the polymer chain axis. However, the collective backbone vibration at 1150 cm^−1^ is likely coupled to bending modes and we cannot make any reliable assumption about its dipole orientation without accurate theoretical modelling.

More detailed discussion is available in the Supporting Information.

**Fig. 4:**
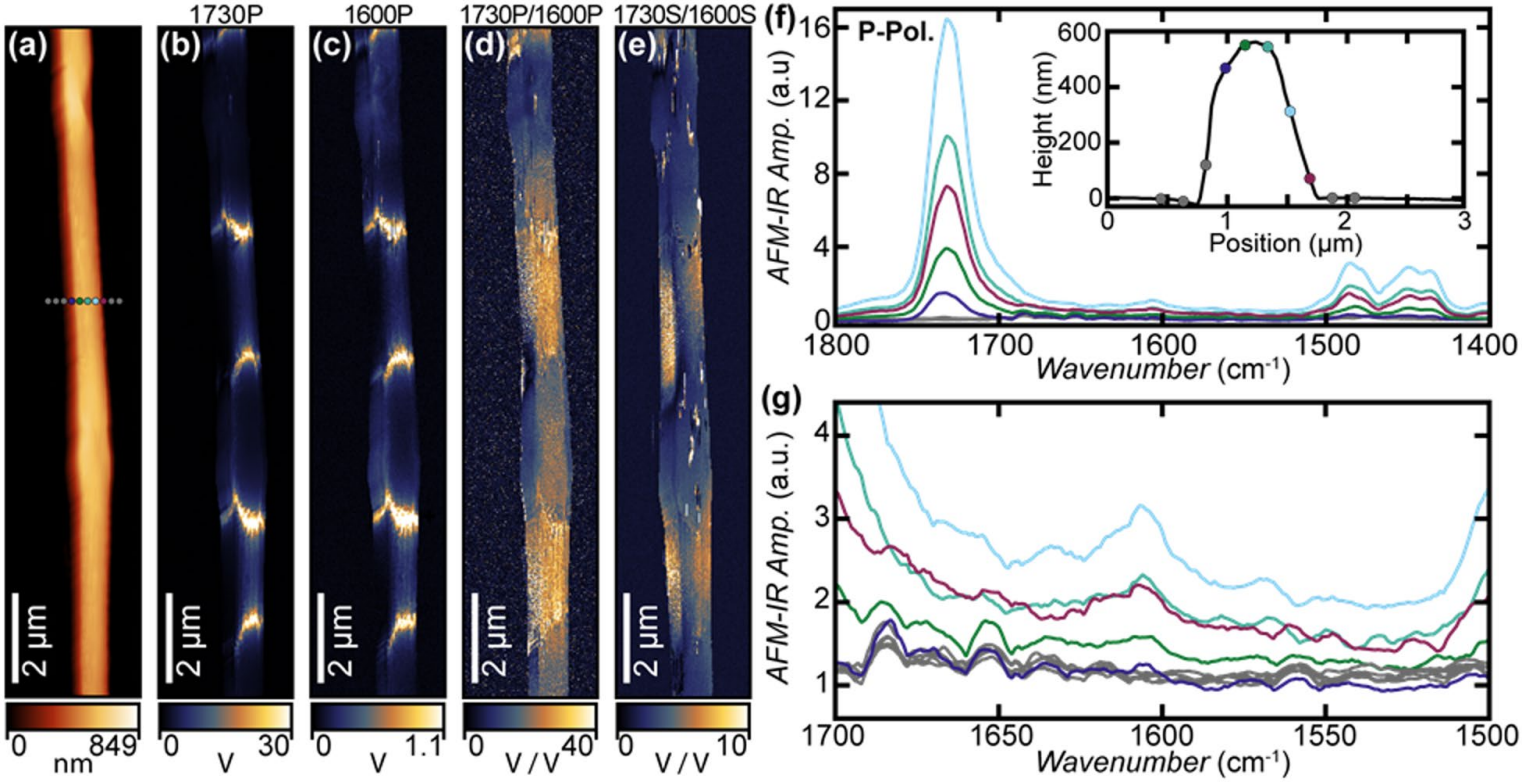
(**a**) AFM topography image and corresponding AFM-IR absorption images (14.6 nm x 23.4 nm pixel resolution in the vertical and horizontal direction, respectively) obtained with p-polarization at (**b**) 1730 cm^− 1^, characteristic of PMMA and (**c**) 1600 cm^− 1^, characteristic of carbyne. AFM-IR image ratios reveal the presence and relative concentration of carbynes (higher relative concentration are represented by blue regions in the fiber) using (**d**) p-polarized and (**e**) s-polarized light. (**f**) P-polarized AFM-IR spectra obtained across the diameter of the fiber, at the region indicated by the colored circles in (a). The inset shows the spectra location plotted on the cross section of the topography at the measured region. (**g**) Zoomed-in p-polarized AFM-IR spectra highlighting the carbyne absorption at 1600 cm^− 1^.

Next, we analyze electrospun PMMA nanofibers loaded with C_4_I (62.5% nominal mass fraction). The AFM topograph (Fig. 4a) shows more obvious local variation in the fiber thickness, i.e., from 490 nm to 670 nm. Despite the high carbyne nominal loading, the PTIR spectra on the fiber are largely dominated by PMMA absorption peaks (Fig. 4f). However, a weak but characteristic peak at 1605 cm^− 1^ (detailed in Fig. 4g) is attributed to the phenyl ring stretching of CAWs included within the fiber^68^. There could be several reasons why this peak appears much weaker than the PMMA peaks. First, the theoretical IR intensity associated with this peak (56 km·mol^− 1^)^68^ is about ten times weaker than the typical carboxylic stretching of esters^81^. Second, the carbyne mechanical properties (e.g., thermal expansion coefficient and stiffness) may differ substantially from the corresponding properties of PMMA, making PTIR signal transduction less efficient in correspondence of the carbyne-rich regions. Detailed discussions of PTIR signal transduction can be found in recent publications^40,48,76^ and reviews^32,33^. Upon first inspection, the PTIR images obtained in correspondence of PMMA (≈ 1730 cm^− 1^, Fig. 4b) and CAWs peaks (≈ 1600 cm^− 1^, Fig. 4c) appear strongly heterogeneous but display a very similar pattern, suggesting that the main features observed are likely related to strong variation in the local mechanical properties (and consequently signal transduction efficiency) rather than composition. Consistently, spectra obtained from the bright and dark regions in Fig. 4b-c show strong variation of intensities but a similar spectral pattern (see Fig. S5). Again, we use image ratios (Fig. 4d, e) to compensate such effect and extract maps more closely attuned to variations in the relative local composition, where blue regions indicate higher relative concentrations of carbynes. The compositional structure of the fibers appears to largely consist of alternating bands of carbyne-rich and PMMA-rich domains ≈ 3 μm long (Fig. 4e, g). The image ratio obtained with s-polarization (Fig. 4e) seems to suggest a larger carbyne concentration in the center of the fiber. The greater variability of the s-/p-polarization intensity ratio values at 1730 cm^− 1^ (≈ 0.50 ± 0.11, see Fig. S6g) measured for another fiber of the same sample, suggests that the orientation of PMMA molecules within this hybrid fiber is more heterogeneous than for PMMA only fibers.

Next, we corroborate the banded compositional structure of these fibers using optical photothermal microscopy (O-PTIR)^82,83^. O-PTIR is another high-resolution IR photothermal technique which uses a visible continuous-wave (CW) laser to probe the photothermal expansion of the sample in place of the AFM cantilever used in AFM-IR. While O-PTIR has a lower spatial resolution (≈ 500 nm) than AFM-IR (≈ 10 nm), it is a non-contact technique and therefore it is less affected by the sample topography and mechanical properties. We note, however, that on this sample, O-PTIR generally has a lower sensitivity than AFM-IR. Therefore, O-PTIR image ratios of sufficient quality were obtained only for fibers with large diameter (> 1 μm) due to the weak IR signal of the carbyne constituent. The O-PTIR spectra are also largely dominated by PMMA absorption peaks (Fig. S7). Interestingly, the O-PTIR ratio map in Fig. S7c clearly qualitatively corroborates the banded compositional structure observed by AFM-IR but reveals longer (≈ 10 μm) domains, perhaps because of the larger size of the measured fiber (≈ 1 μm). However, neither AFM-IR nor O-PTIR signal intensities were sufficiently strong to quantitatively determine the orientation of the carbyne molecules within the fibers.

**Fig. 5:**
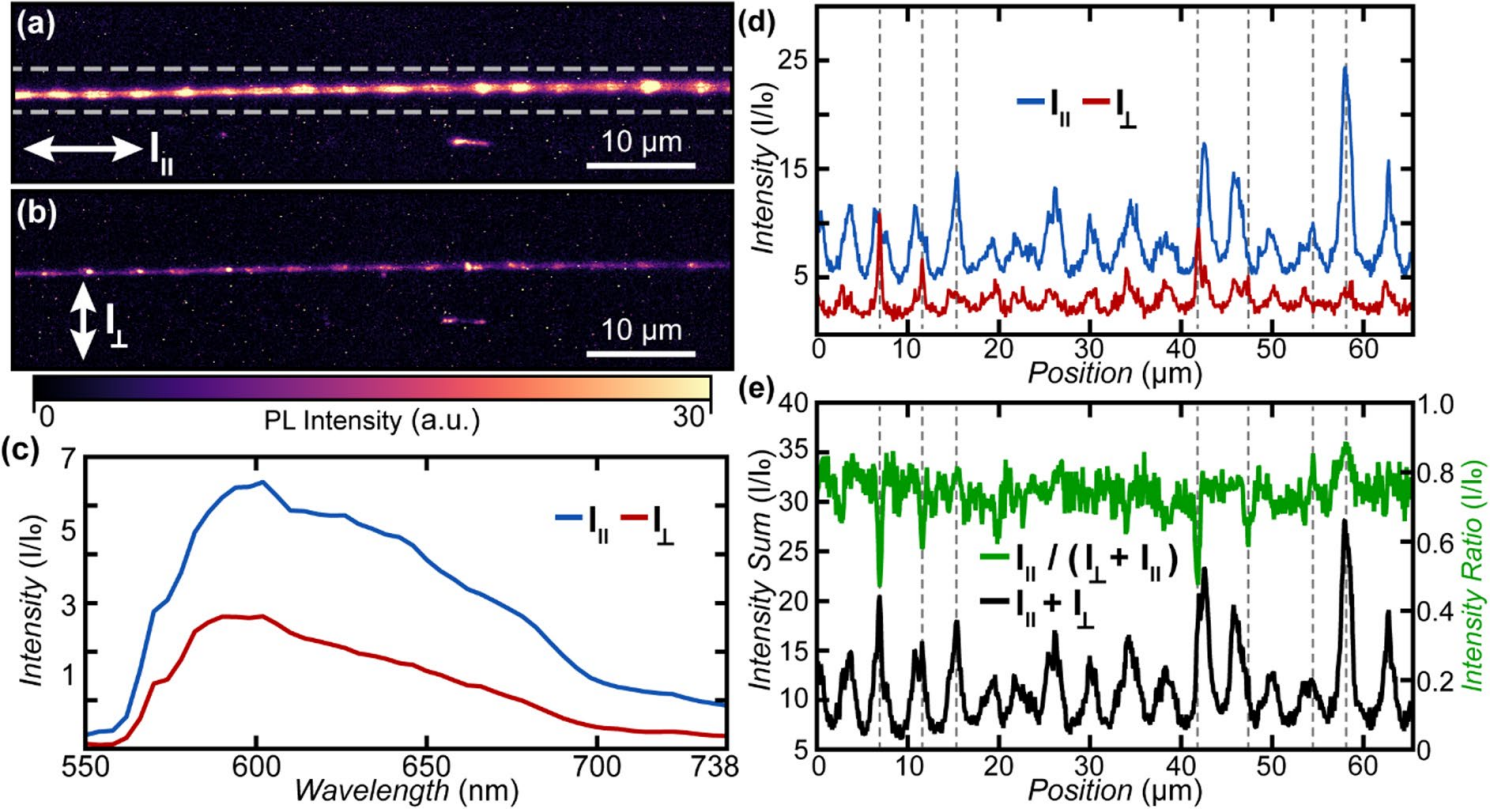
PL intensity maps of a CAW-loaded PMMA nanofiber obtained with linearly polarized laser excitation (532 nm) and analyzer both aligned either parallel (**a**) or perpendicular (**b**) to the fiber axis. (**c**) PL spectral intensity (integrated over the marked region in (a)) obtained with linearly polarized excitation and analyzer both either parallel (blue) or perpendicular (red) to the fiber axis. (**d**) PL intensity profiles at 602 nm emission along the center of the fiber obtained with linear polarization either parallel (blue) or perpendicular (red) to the fiber axis. (**e**) Sum (black) and ratio (green) profiles of the PL intensities along the center of the fiber obtained with linearly polarized light either parallel and perpendicular to the fiber axis. We use the PL sum profile as a proxy for the relative carbyne concentration and the PL ratio profile as a proxy for relative variations of carbyne orientation.

To investigate the orientation of the carbyne-rich domains, we employed polarized hyperspectral PL (see Fig. 5)^36,37^. Two sets of experiments were conducted: one with the linearly polarized excitation laser and analyzer aligned parallel to the fiber axis (Fig. 5a, hereafter called parallel PL, analogous to s-polarization in the AFM-IR experiments) and one with the laser polarization and the linear analyzer positioned perpendicular to the fiber axis (Fig. 5b, hereafter called perpendicular PL, analogous to p-polarization in the AFM-IR experiments). Since PMMA provides a negligible contribution to the PL intensity, the PL images encode the distribution and orientation of the CAW component within the fibers which corroborate the compositionally heterogeneous band structure observed by AFM-IR and O-PTIR. We use the sum of the PL intensities obtained with polarization parallel ($\:{I}_{\parallel\:}$)and perpendicular ($\:{I}_{\perp\:}$) to the fiber axis as a proxy to estimate the relative distribution of the carbynes within the fiber, highlighting 3 μm to 4 μm wide bands along the fiber axis (Fig. 5e, black). The PL intensity derives from the CAW component and generally shows stronger intensity for light polarization parallel to the fiber axis (Fig. 5a, c,d), suggesting that CAWs in the fiber are predominantly aligned along the fiber axis. We use the ratio of the PL intensities $\:{I}_{\parallel\:}/{(I}_{\parallel\:}+{I}_{\perp\:})$ as a proxy, to estimate the relative orientation distribution of carbynes within the fiber (Fig. 5e, green). Since $\:{I}_{\parallel\:}/{(I}_{\parallel\:}+{I}_{\perp\:})>0.5$ it appears that the CAWs are generally well aligned along the fiber but display some alignment heterogeneities, highlighted by peaks and dips in the $\:{I}_{\parallel\:}/{(I}_{\parallel\:}+{I}_{\perp\:})\:$trace (green in Fig. 5e) representing 1.5 μm to 3 μm wide regions with increased and decreased orientation, respectively.

## Conclusions

In this study, we used high spatial resolution polarization-sensitive spectroscopic techniques to study the compositional heterogeneity and molecular orientation anisotropy of electrospun PMMA nanofibers loaded with carbon molecular wires at the ≈ 20 nm (AFM-IR) and at the ≈ 500 nm (O-PTIR, hyperspectral PL) scales. In summary, this complex characterization at the single nanofiber level, highlights the effectiveness of electrospinning for producing anisotropic nanocomposites, not only for the polymeric fibers but also for molecular additives just four carbon atom long.

Careful calibration of polarized AFM-IR spectra, accounting for the polarization dependent reflectivity of the mirrors in the AFM-IR setup and for the tip enhancement of the AFM probe, enables assessment of the molecular orientation of polymer chains at the nanoscale. For unloaded PMMA fibers, AFM-IR image ratios reveal a homogeneous, uniaxial orientation of the polymer chains along the fiber axis. Semiquantitative analysis of the experimental C = O stretching (≈ 1730 cm^− 1^) dichroic ratio in PMMA restricts the angle between the C = O moiety and the polymer backbone to a range from 67.2° to 90°. Although this model-free analysis does not provide a means to extract a distribution for the molecular chain orientation, the nearly orthogonal arrangement of the functional group and a highly planar polymer backbone resulting from this analysis match the expectations of the electrospinning process and agree with prior statistical analysis of molecular dynamics. We propose to describe the molecular alignment of the PMMA fiber using the average value for the allowed C = O angles ($\:{\psi\:}_{avg}=78.6^\circ\:$) which implies an average Herman’s function $\:{f}_{avg}=0.623$ and an average angle between the PMMA chains and the fiber axis of $\:{\stackrel{-}{\varphi\:}}_{avg}=30.1^\circ\:.$.

Notably, AFM-IR, O-PTIR, and hyperspectral PL images consistently show that the incorporation of CAWs into the PMMA fibers results in a heterogeneous (banded) composition with alternating PMMA-rich and CAW-rich regions (3 μm to 4 μm wide) along the fiber axis. Furthermore, hyperspectral PL maps selectively displaying the emitted intensity from CAW molecules reveal that the CAWs molecules are predominantly aligned along the fiber axis direction, with locally sparse heterogeneities in the molecular orientation occurring in regions 1.5 μm to 3 μm wide. These results indicate that electrospinning is an effective process to impart molecular orientation of both polymer matrix and of molecular additives, even for the short molecules employed here. Overall, we envision that the electrospinning process, coupled with the widely applicable, composition and molecular orientation sensitive techniques used in this work will facilitate the rational design and realization of anisotropic functional nanocomposites with tunable optical and electronic properties. While this goal is conceptually feasible, efforts to correlate processing parameters (e.g. flow rate, electric field, etc.) to tailored anisotropic fiber structures will strongly benefits from efforts aiming at increasing the measurement throughput of AFM-IR^57^ and O-PTIR^84^.

## Supplementary Information

Below is the link to the electronic supplementary material.

Supplementary Material 1

## Data Availability

The data that support the findings of this study are available from the corresponding author upon reasonable request.
