# Supplementary material for: Nanoscale mapping of composition and orientation in electrospun polymeric nanofibers loaded with carbon atomic wires
Source: Sci Rep. 2026 Apr 4;16:16247. doi: 10.1038/s41598-026-46988-8 (PMC13201752; doi:10.1038/s41598-026-46988-8)
Supplement: Supplementary file 1 — Supplementary Material 1 [file 41598_2026_46988_MOESM1_ESM.docx]

**Supporting Information**

Nanoscale Mapping of Composition and Orientation in Electrospun Polymeric Nanofibers Loaded with Carbon Atomic Wires

Simone Melesi^a^, Devon S. Jakob^b^, Jeremy F. Schultz^b^, Adam J. Biacchi^b^, Piotr Pińkowski, Bartłomiej Pigulski, Chiara Castiglioni^c^, Chiara Bertarelli^c^, Sławomir Szafert^d*^, Carlo S. Casari^a*^, Andrea Centrone^b*^

1. Micro and Nanostructured Materials Laboratory — NanoLab, Department of Energy, Politecnico di Milano, Via Lambruschini 8, Milano, 20156, Italy
2. Nanoscale Device Characterization Division, Physical Measurement Laboratory, National Institute of Standards and Technology, Gaithersburg, Maryland, 20899, United States
3. Department of Chemistry, Materials and Chemical Engineering “Giulio Natta”, Politecnico di Milano, Milano 20133, Italy
4. Faculty of Chemistry, University of Wrocław, 14F. Joliot-Curie, 50-383 Wrocław, Poland

**Table of Contents:**

**Figure S1:** Chemical structure of C_4_I.

**Figure S2:** Schematic of the electrospinning setup

**Figure S3:** Single-wavelength S- and P-polarized AFM-IR images of non-loaded PMMA fiber at 1150 cm^-1^ and 1730 cm^-1^.

**Figure S4:** AFM-IR Spectra correction and normalization.

**Figure S5:** AFM-IR Spectra complementing Figure 4 of the main text

**Figure S6:** AFM-IR Spectra on CAW loaded PMMA fiber, additional dataset

**Figure S7:** O-PTIR image ratios.

**Figure S8:** Topography and AFM-IR profiles for carbyne-loaded PMMA fibers highlighting banded PMMA-rich domains.

**Detailed Discussion of the PMMA Molecular Structure**

**
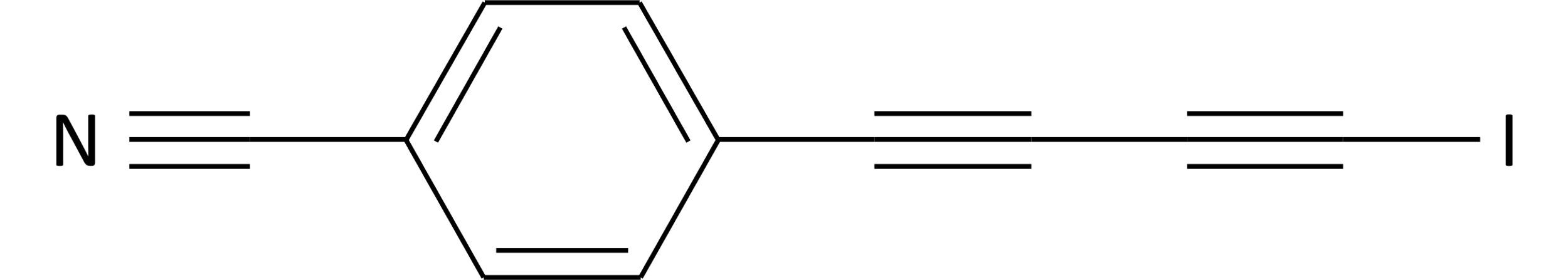
**

**Figure S1:** Chemical structure of C_4_I.


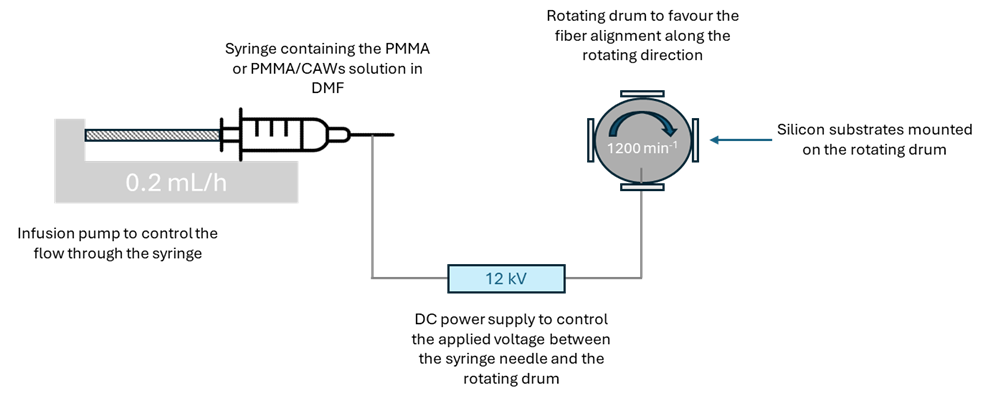


**Figure S2:** Schematic representation of the electrospinning setup equipped with a rotating drum used to produce aligned electrospun nanofibers.


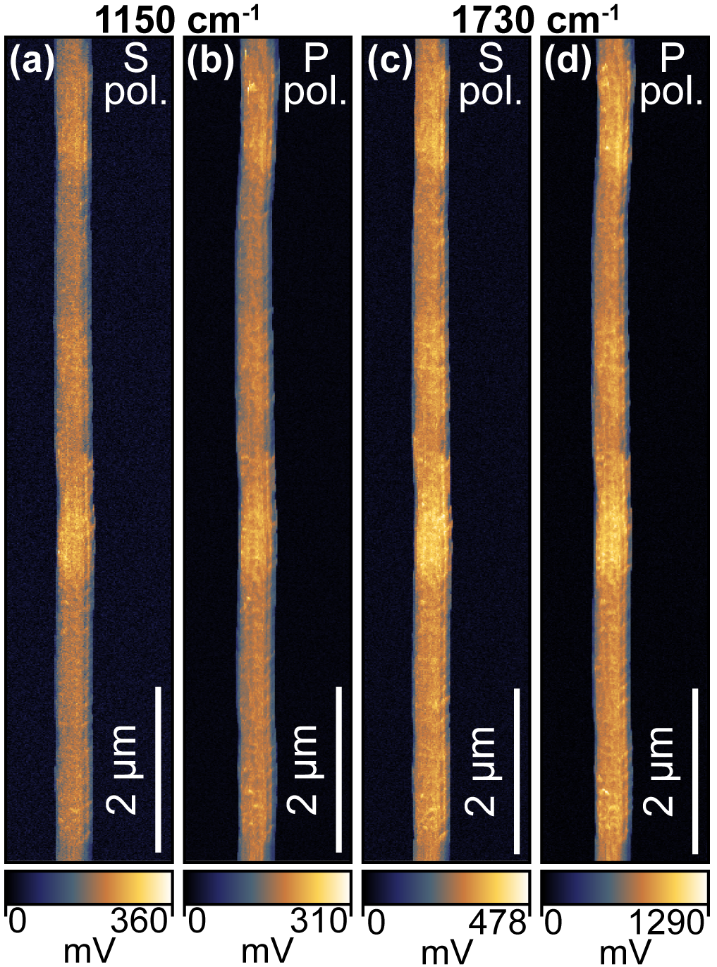


**Figure S3:** AFM-IR absorption images (9.8 nm x 15.6 nm pixel resolution in the vertical and horizontal direction, respectively) obtained at 1150 cm^-1^, corresponding to the skeletal stretching of PMMA, with **(a)** s-polarized light and **(b)** p-polarized light. AFM-IR absorption images (9.8 nm x 15.6 nm pixel resolution in the vertical and horizontal direction, respectively) obtained at 1730 cm^-1^, corresponding to the carboxylic stretching peak of PMMA, with **(c)** s-polarized and **(d)** p-polarized light. The scale bar is 2 µm.


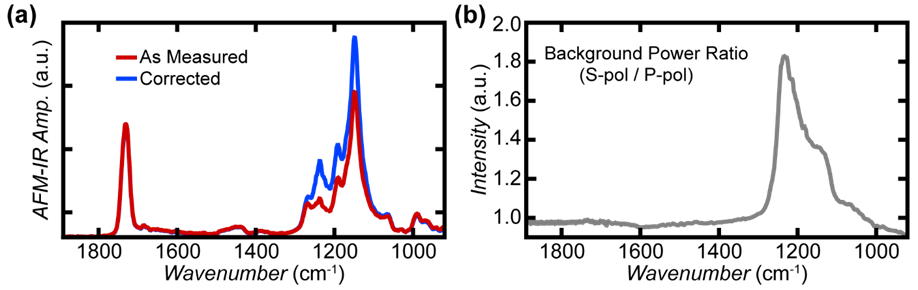


**Figure S4: (a)** AFM-IR s-polarization spectrum of the PMMA fiber in Fig. 2e as measured (red) and corrected to account for p- and s-polarized background intensity differences due to polarization-dependent reflectivities of the protected silver mirrors used in the AFM-IR setup(blue). **(b)** P-polarization and s-polarization laser background intensity ratio.


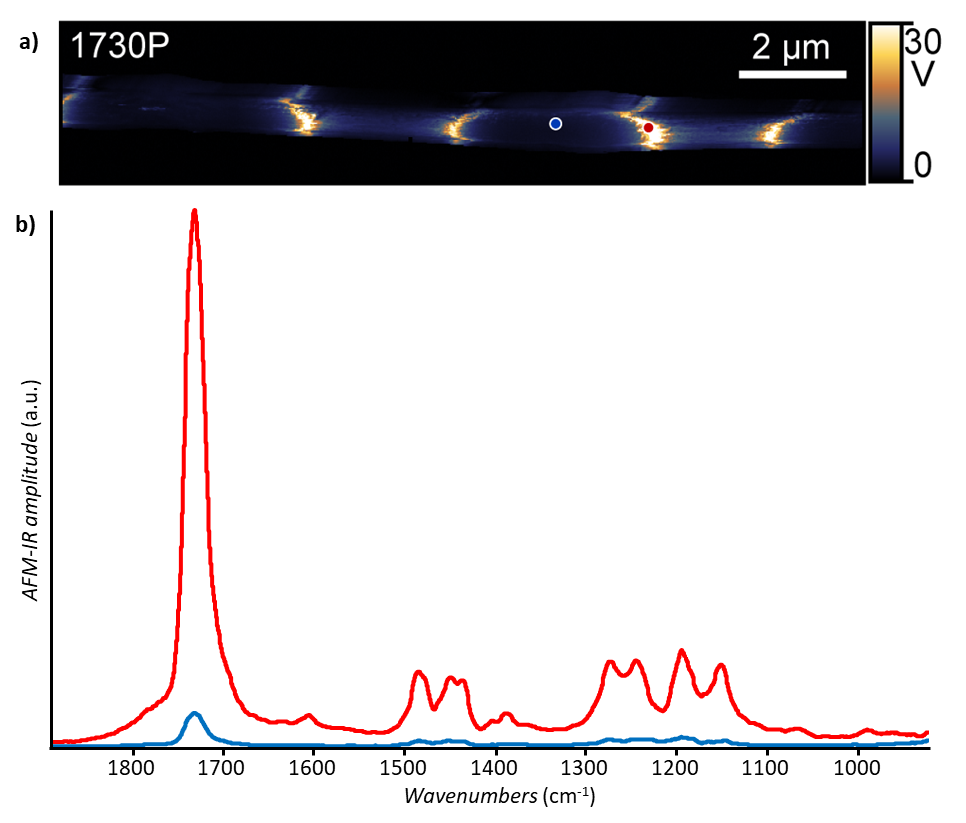


**Figure S5: (a)** AFM-IR absorption image (14.6 nm x 23.4 nm pixel resolution in the horizontal and vertical direction, respectively) obtained at 1730 cm^-1^ with p-polarization; same as Figure 4b. **(b),** AFM-IR p-polarization spectra obtained at the color-coded locations in panel (a).


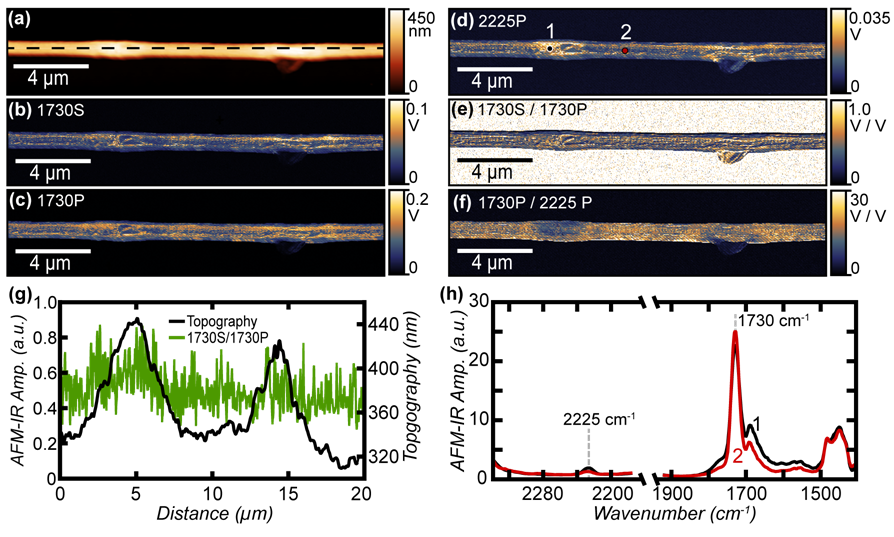


**Figure S6: (a)** AFM topography image and corresponding AFM-IR absorption images (19.5 nm x 31.2 nm pixel resolution in the horizontal and vertical direction, respectively) obtained with **(b)** 1730 cm^-1^ s-polarization, **(c)** 1730 cm^-1^ p-polarization, **(d)** 2225 cm^-1^ p-polarization. **(e)** AFM-IR absorption ratio images measured with s- and p-polarization at 1730 cm^-1^ detailing slight variations PMMA chain alignment. **(f)** AFM-IR absorption ratio images (1730/2225) measured p-polarization detailing the relative distribution of carbynes (higher relative concentration are represented by blue regions in the fiber). **(g)** AFM topography (black) and AFM-IR intensity ratio (s-/p-polarization) at 1730 cm^-1^ (green) along the fiber axis in correspondence to the line marked in panel (a). **(h)** AFM-IR spectra obtained the color-coded locations in panel d. This dataset was obtained using a QCL emitting from 1400 cm^-1^ to 1915 cm^-1^ and from 2175 cm^-1^ to 2339 cm^-1^.


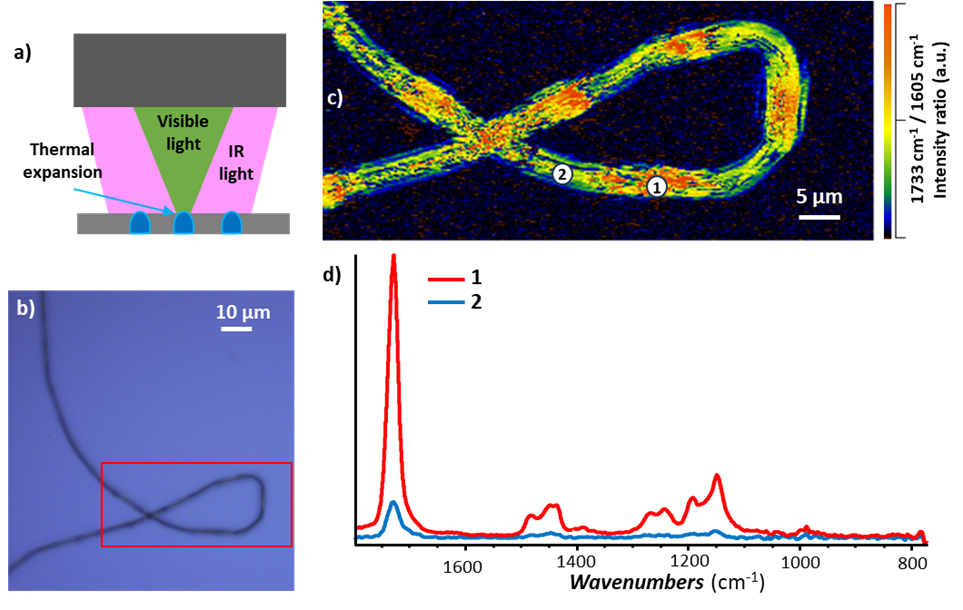


**Figure S7:** **(a)** O-PTIR schematic. **(b)** Optical image of carbyne-loaded PMMA fiber. The red box delimits the measured in panel-c. **(c)** O-PTIR intensity map ratio (1605 cm^-1^ / 1733 cm^-1^) showing PMMA-rich areas (orange) and carbyne-rich areas (green and yellow). **(d)** O-PTIR spectra obtained at marked locations in (c).


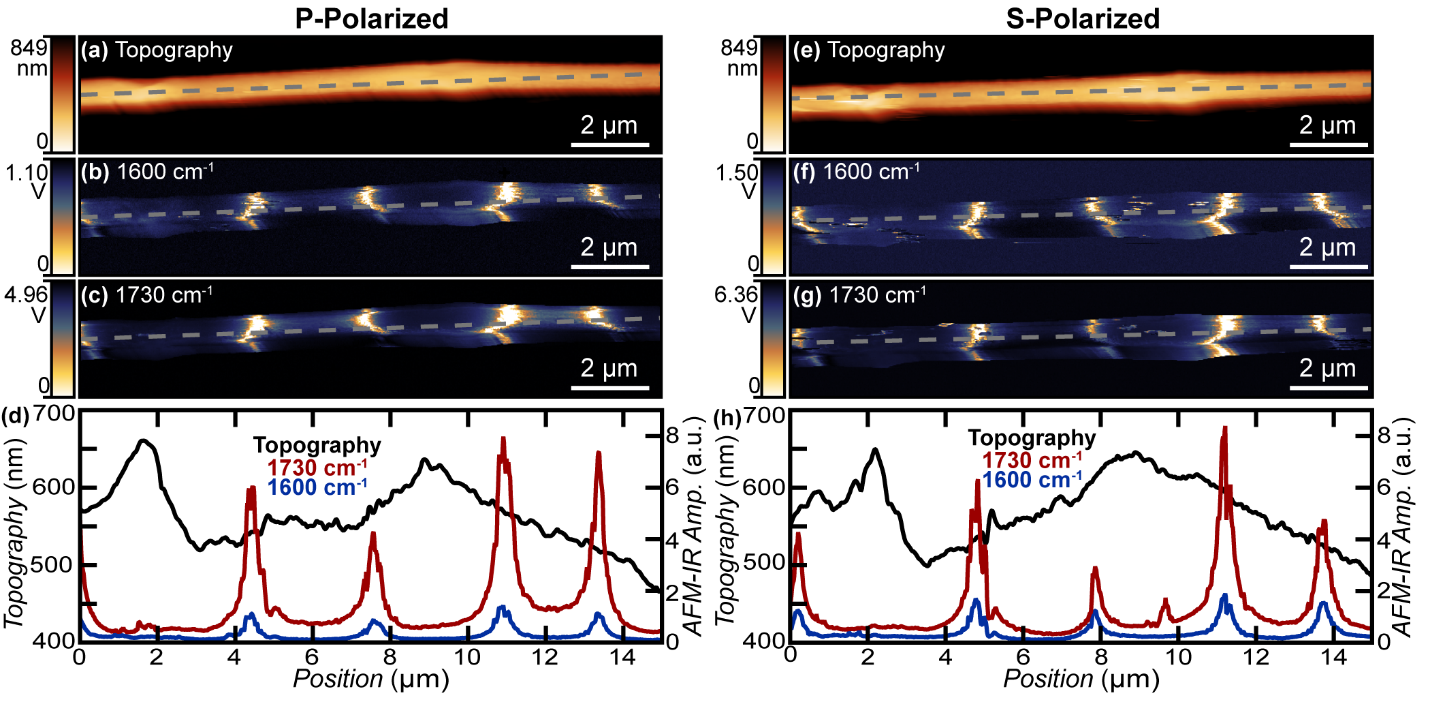


**Figure S8:** AFM-IR topography and absorption images (23.4 nm x 14.6 nm pixel resolution in the vertical and horizontal direction, respectively) of carbyne-loaded PMMA fibers. Measurements are obtained with both **(a-d)** p-polarized and **(e-h)** s-polarized light. (**d, h**) Line profiles of topography, AFM-IR absorption at 1600 cm^-1^ (carbyne), and AFM-IR absorption at 1730 cm^-1^ (PMMA) in correspondence to the grey dashed lines in panels a-c and e-g, respectively. The scale bars are 2 µm.

**Detailed Discussion of the PMMA Molecular Structure**

**PMMA Structure and Definition of the Polymer Chain Axis**

The determination of the polymer orientation in a sample characterized by uniaxial orientation, (e.g., a fiber) requires definition of the director (**D**) and the chain axis (**z**). The Hermans function $f$provides information on the second momentum of a probability distribution function, $\mathcal{F(}\cos\left( \phi\right))$, where $\phi$ is the angle between **D** and the chain axis **z** of a polymer chain. **D** coincides with the fiber axis, while the definition of the fiber axis is less trivial in the case of an amorphous polymer. We assume that the electrospinning procedure results in extended chain conformations of the polymer molecules, so that we can identify a chain axis for long sequences of PMMA units. As discussed in more detail below, we expect that the all-trans conformation of the polymer backbone will be favored since it corresponds to the most elongated structure – i.e. to the largest end-to-end distance of the chain. In this case, the direction of the chain axis is the straight line joining equivalent carbon atoms of the backbone (see Fig. 3a).

**C=O Stretching Dipole Moment Direction and Structural Models**

The direction of the transition dipole associated to the C=O stretching band at 1730 cm^-1^ can be inferred based on a model structure of PMMA under the hypothesis that the C=O stretching modes are localized vibrational modes, each of them involving the stretching of individual C=O bonds, analogously to a diatomic molecule. In this case, the C=O stretching dipole derivative is parallel to the C=O bond and the intensity of the band at 1730 cm^-1^ is determined by the vibrational transition moment of individual oscillators characterized by a $\frac{\partial\vec{M}}{\partial Q}$ vector which forms with the chain axis (z), an angle $\psi$ identical to the angle between the C=O bond direction and $\boldsymbol{z}$. According to the above assumption we can simply focus on the direction of the C=O bond.

The orientation of the C=O bond of PMMA depends on the several conformational degrees of freedom (torsional angles) which can affect its geometry. Each PMMA chemical unit exhibits two torsional angles along the polymer backbone (θ_1_ and θ_2_) and a torsional angle (θ_3_) which describes the rotation of the COOCH_3_ group around the C-C bond which links it to the chain (see Fig. 3b).

An additional complication arises from the fact that atactic polymers (as the a-PMMA in this work) are made by sequences of units with different configurations – namely, isotactic (i-PMMA) or syndiotactic (s-PMMA) sequences - which could affect the chain conformation. According to X-ray diffraction (XRD) studies on semi-crystalline samples of stereo-regular i-PMMA and s-PMMA, the chain conformation in the crystalline domains is described as a regular helix. [1–3] On the other hand, stretch-oriented chains of the amorphous atactic polymer (a-PMMA) should preferentially take extended conformations, possibly close to the all-trans conformation of the chain (θ_1_ ≅ θ_2_ ≅ 180°), corresponding to the largest end-to-end distance, which complies with highly stretched polymer chains because of electro-spinning. This hypothesis finds support in Behbahani et al., where the structure of amorphous PMMA chains with different tacticity is discussed by means of molecular dynamics simulations followed by a statistical analysis. [4] Even in absence of applied stress, the authors conclude that the “preferred” conformation of the chains is the all-trans structure, which confirms the robustness of the hypothesis of an all trans, planar backbone structure.

Behbahani et al. reports that the most probable θ_3_ values are θ_3_ = 0° and θ_3_ = 180° have the ester group in a plane (α) perpendicular to the plane containing the all-trans planar polymer backbone (plane γ). [4] In this case, the C=O bond and any vector parallel to the C=O bond direction is perfectly orthogonal to the chain axis, namely $\psi=90^{\circ}$ (This structure is illustrated in Fig. 3). In the hypothesis that θ_3_ should take the same value in all PMMA units, this choice minimizes the repulsive interactions due to the steric hindrance of the COOCH_3_ groups belonging to adjacent units.

However, if each unit is allowed to freely choose the θ_3_ value to better accommodate the bulky lateral groups, or if the steric hindrance of the side groups vary along the chain according to the disordered tacticity, deviations from $\psi=90^{\circ}$ should be expected. In this case, the C=O bonds belonging to different units could show a component parallel to the $z$axis, namely a $\psi$ value smaller than 90°. The value θ_3_ = 90° (i.e., the α plane parallel to the γ plane) gives the largest parallel component of the C=O bond direction, with $\psi=30^{\circ}.$ However, it is easy to realize that values of θ_3_ close to 90° are far from being acceptable because of the steric hindrance of the lateral groups. Therefore, we can reasonably conclude that there is a limited interval of “accessible” $\psi$ values, close to $90^{\circ}$, in very good agreement with the suggestion obtained from the experimental dichroic ratio: $67.2^{\circ}\leq\psi\leq90^{\circ}$.

**References**

[1] A.J. Christofferson, G. Yiapanis, J.M. Ren, G.G. Qiao, K. Satoh, M. Kamigaito, I. Yarovsky, Molecular mapping of poly(methyl methacrylate) super-helix stereocomplexes, Chem Sci 6 (2015) 1370–1378. https://doi.org/10.1039/C4SC02971B.

[2] H. Kusanagi, Y. Chatani, H. Tadokoro, The crystal structure of isotactic poly (methyl methacrylate): packing-mode of double stranded helices, Polymer, 35 (1994) 2028–2039.

[3] H. Tadokoro, Y. Chatani, H. Kusanagi, M. Yokoyama, Structure of Isotactic Poly (methyl methacrylate), Macromolecules 3 (1970) 441–447.

[4] A.F. Behbahani, S.M.V. Allaei, G.H. Motlagh, H. Eslami, V.A. Harmandaris, Structure and dynamics of stereo-regular poly (methyl-methacrylate) melts through atomistic molecular dynamics simulations, Soft Matter 14 (2018) 1449–1464.
